# Forward Planning: A Staffing Framework and Ratios for Psychosocial Oncology and Supportive Care Hiring Practices as Cancer Care Models Evolve

**DOI:** 10.3390/curroncol33050290

**Published:** 2026-05-14

**Authors:** Carole Mayer, Marianne Arab, Kimberley Thibodeau, Celestina Martopullo

**Affiliations:** 1 Health Sciences North Research Institute, Sudbury, ON P3E 2H3, Canada; 2 Division of Psychosocial Oncology, Cumming School of Medicine, University of Calgary, Calgary, AB T2N 4N1, Canada; celestina.martopullo@albertahealthservices.ca; 3 Nova Scotia Health, Cancer Care Program, Psychosocial Oncology Program, Halifax, NS B3H 2Y9, Canada; marianne.arab@nshealth.ca; 4 Psychosocial Oncology Program, McGill University Health Center, Montréal, QC H4A 3J1, Canada; kimberley.thibodeau@muhc.mcgill.ca; 5 Department of Psychosocial Oncology, Arthur J.E. Child Comprehensive Cancer Centre, Cancer Care Alberta, 3395 Hospital Drive NW, Calgary, AB T2N 5G2, Canada

**Keywords:** staffing framework, staffing ratios, psychosocial oncology, supportive care, innovative cancer care models, social worker, psychologist, spiritual care practitioner, physiotherapist, dietitian, occupational therapist, interdisciplinary team, workload measurement, patient-reported outcome measures, cancer policy

## Abstract

Innovative models of cancer care have emerged in response to advances in cancer treatment, expanding technologies that bring care closer to home and address COVID-19-related challenges and concerns about a shrinking healthcare workforce. Despite the advancements made, the psychosocial impact on people affected by cancer persists. The psychosocial burden of cancer underlines the need for patient access to evidence-based psychosocial oncology (PSO) and supportive care (SC) interventions. As models of care evolve, hiring practices of PSO professionals must also evolve for cancer patients to access properly staffed PSO programs that deliver high-quality and efficient services. In 2019, the Canadian Association of Psychosocial Oncology (CAPO)–Clinical Advisory Committee consulted administrators and clinicians across Canada to understand caseload volumes of PSO professionals with a goal to set staffing ratios. The engagement process revealed that there is no consistency in staffing PSO programs across Canada, let alone staffing ratios for PSO disciplines. In 2022, CAPO introduced a 10-point staffing framework and formula to calculate staffing ratios for hiring PSO professionals, beginning with the social work discipline. The goal of this paper is to provide updates to the existing framework and demonstrate how the formula can be adapted to other PSO disciplines. To our knowledge, this is the first published paper in Canada outlining the calculations for a PSO staff framework and formula. The authors advocate for greater transparency when reporting PSO staffing ratios across Canada, using this framework as a reference point. Organizations reporting on the cancer system performance are encouraged to develop PSO indicators, starting with tracking patient access to PSO services.

## Simple Summary

The delivery of cancer treatment is constantly evolving, driven by new research, technologies that bring services closer to home, and the need to address healthcare staffing shortages. What has not changed is the emotional, psychological, and social impact on people diagnosed or affected by cancer. In this paper, we describe how to develop a staffing plan and apply a formula to calculate the appropriate number of healthcare professionals needed to provide specialized psychosocial and rehabilitative care. Originally introduced by the Canadian Association of Psychosocial Oncology in 2022, based on the discipline of social work, this paper reports on updates made to the staffing plan and formula. We also explain how to adapt these tools to other disciplines providing specialized psychosocial and supportive care services. Finally, we emphasize the need to develop performance indicators to measure and improve patient access to specialized support services across Canada.

## 1. Introduction

Various discoveries relevant to cancer drug development, immunotherapy, targeted interventions, and precision medicine have contributed to the progress made in diagnosing and treating cancer. Similarly, telehealth for cancer care and provision of Psychosocial Oncology (PSO) services have expanded considerably over the past decade [1,2,3]. Its use increased sharply across cancer programs during the COVID-19 pandemic [4,5], as the psychosocial burden on patients intensified [6,7,8,9,10,11,12]. There was a need for ongoing adaptations in care delivery models for continuity of cancer care [13,14,15]. Today, telehealth and other modes of virtual care continue to support the delivery of cancer treatment, clinical trials, and care closer to home, particularly for people living in remote and rural communities [4,5]. Despite these clinical and technological innovations, cancer patients continue to experience the acute and long-term psychosocial burden of cancer morbidity [16,17]. Without a stable and adequately staffed workforce, the psychosocial needs of people affected by cancer remain insufficiently addressed. The purpose of this paper is to present the updates to the original staffing framework and formula released by CAPO in 2022 for the discipline of social work [18] and demonstrate how to adapt the framework and calculations to other PSO disciplines.

## 2. Background

Psychosocial oncology is a specialization in cancer care focused on treating the social, psychological, emotional, spiritual, quality-of-life, functional, and practical aspects of cancer, starting from prevention through bereavement [19]. Developing a staffing formula for PSO professionals is challenging, due to limited policy and research papers available describing how staffing ratios are determined and calculated. Similar gaps exist for healthcare professionals working in the medical system.

A systematic review focused on identifying staff ratios for nine allied health care disciplines to determine their usefulness for human resources planning [20]. The disciplines were audiology, dietetics and nutrition, exercise physiology, occupational therapy, podiatry, physiotherapy, psychology, social work, and speech pathology [20]. Twelve papers met the study criteria for inclusion of the review [20]. The authors conclude that research on staffing ratios for these nine disciplines is limited and lags behind other disciplines such as nursing and medicine [20].

Two papers specific to PSO social work were referenced in developing the original CAPO staffing framework and formula [18] to further elaborate the breakdown of calculations for staffing ratios. A U.S. study reported that a PSO social worker could manage approximately 10–12 highly distressed cancer patients per month, based on estimates provided by PSO Department heads [21]. This amounts to a caseload of approximately 120 cancer patients per year while still fulfilling responsibilities such as psychoeducation, case management, group work, advocacy, and other assigned duties [21]. The authors estimated that 30% of cancer patients experience high distress, based on previous study findings [21]. Therefore, a cancer program admitting 3600 new cancer cases annually would require 9.0 full-time equivalent (FTE) social workers to support 1080 highly distressed cancer patients [21]. The limitation of this study is that the authors do not describe the patient population found to be “highly distressed” as part of a caseload. The breakdown of hours to meet the needs of the patient population is missing, making it difficult to understand the amount of time spent on direct patient care activities and non-patient care activities.

A Canadian study differentiated PSO social work roles into resource social workers and clinical social workers, describing responsibilities and required education levels [22]. They also described the roles of the social workers based on the different cancer care setting, including tertiary cancer centers, regional cancer centers, and community clinics [22]. In tertiary cancer centers, resource social workers focused specifically on improving patients’ access to resources, while clinical social workers provided clinical counseling/psychotherapy. Social workers in regional cancer centers typically performed both functions to support patients in accessing resources and provided counseling/psychotherapy. Social workers working in community cancer centers delivered similar services via telephone and/or telehealth [22]. Reported staffing levels included 12.0 FTE social workers across two tertiary sites; 4.0 FTE social workers across four regional sites; and 1.3 FTE social workers across 11 community sites [22]. The authors do not describe how the staffing ratios were determined [22], leaving it unclear whether the staffing ratios reflect patient needs and/or availability of funding.

Administrators might find it difficult to apply the staffing ratios presented in these two papers. One paper [21] estimates the volume of a social worker’s caseload based on the level of distress of the cancer patient population served and the number of cases that are manageable as part of a social worker’s caseload. The other paper [22] reports staffing ratios based on assigned responsibilities for doing counseling/psychotherapy vs. advocacy and instrumental work based on cancer setting. Nonetheless, both papers highlight the complexity of establishing PSO staffing ratios.

In 2022, the CAPO Clinical Advisory Committee released a 10-point framework and formula, endorsed by PSO clinical leaders from across Canada, to calculate staffing ratios for hiring PSO professionals, beginning with the social work discipline [18]. The work evolved from a 2019 consultation process with administrators and clinicians working in various capacities across cancer programs in Canada. The feedback received highlighted discrepancies across provinces for the various PSO disciplines, with no clear rational for how staffing levels are achieved. Documents outlining the scope of practice of PSO professionals were instrumental in developing the staffing framework, focusing on the PSO discipline-specific responsibilities, and the credentialing requirements [19,23,24]. The formula to calculate staff ratios was developed based on administrative data shared by members of the expert panel and drawing on clinical and administrative expertise to achieve consensus.

This article provides updates to the existing CAPO staffing framework and formula, to support staffing ratio calculations for other PSO disciplines. Administrators can follow steps 1 and 2 to assess for strengths and gaps within their PSO program, prior to moving forward with hiring discipline-specific PSO providers. The framework also supports the development of PSO models of care that align with patient needs while ensuring manageable workloads. (For the purpose of this paper, “patient” refers to an individual diagnosed with cancer, and “family member” refers to a patient’s closest support network defined by the patient; patients and family members may access PSO services for emotional, psychological, social, instrumental, and/or spiritual support. For ease of reading, we only reference patient(s), which includes family member(s).) Staffing ratios include available work hours, further divided by allocated time across core responsibilities. As such, the formula offers an objective methodology for calculating the number of positions required by PSO disciplines. These ratios serve as reference points for PSO programs across Canada, including when reporting, adapting, and comparing PSO staffing models. If staffing variations exist across programs and provinces, they can be explained by making the calculations transparent using the framework and staffing formula.

## 3. PSO Staffing Ratio Framework

### 3.1. Step 1: Hiring Principles

Program setting: Reviewing the program setting and infrastructure is the first planning step for hiring PSO professionals. Practicing in an outpatient tertiary cancer center differs from practicing in a hospital setting or community clinic, with variations in scope of practices [22] and allocation of time allowed for different responsibilities assigned to PSO professionals [18]. Identifying members of the PSO interdisciplinary team (e.g., psychiatrists, psychologists, social workers, spiritual care practitioners, dietitians, physiotherapists, occupational therapists, etc.), as well as the broader cancer care team (e.g., oncologists, nurses, radiation therapists, pharmacists, etc.) provides clarity regarding the range of professional roles required to meet the needs of people affected by cancer. Cancer programs affiliated with academic centers have implications for PSO roles and responsibilities. PSO professionals are perhaps engaged in teaching, supervising students, conducting research, and participating in committee work. PSO professionals may hold administrative leadership responsibilities, such as planning, developing, implementing, and evaluating new programs. PSO professionals, working in regional cancer centers and/or community clinics, often consult PSO team members in tertiary cancer centers who provide mentoring, case consultation, one-on-one education, and general guidance. These responsibilities impact time spent on direct clinical practice, and must be considered during workforce planning.

Program Infrastructure: The work environment shapes the roles and responsibilities of PSO professionals. A well-developed infrastructure supports PSO professionals in practicing at their highest level of competency and in accordance with professional standards. The presence of effective change management processes supports efficiency and innovation in the organization. When PSO programs have well-defined position statements, standards of care, policies, and procedures aligned with organizational values and mission statements, it clarifies the recruitment strategy to attract and hire the best candidates.

Administrative considerations: Maximizing PSO work time for direct clinical service depends on efficient processes and support systems. Triage protocols to assess the urgency of referrals, on-call rosters for designated PSO professionals to respond to daily urgent referrals, digital referrals linked to a digital charting system, and a digital workload measurement system all contribute to improve efficiencies and reduced administrative workloads. Adequately resourced administrative support staff reduce administrative burdens placed on PSO professionals who can then focus their time on patient care activities. Administrators are then in a much better position to effectively track, analyze, measure, and report patient outcomes.

Clinical considerations: From a clinical perspective, understanding the cancer populations receiving treatment is essential for determining the specialized knowledge and skills required of PSO professionals. Factors such as the cancer trajectory (prevention, pre-diagnosis, diagnosis, treatment, rehabilitation, survivorship, recurrence, palliative care, end of life care and bereavement), demographic, bio-psycho-social-spiritual-practical/instrumental characteristics, and needs of specific cancer patient populations all determine how PSO clinicians provide patient-centered care. The format of service delivery is another important consideration. For example, social workers and psychologists may provide individual, couple, family, and group interventions, whereas dietitians may focus their interventions more specifically on patients and caregivers. PSO services may be delivered in person, by telephone, or virtually, and in some cases, protocols permit communication with patients via texts or email (e.g., for head and neck patients with limited verbal communication capacity).

The use of patient-reported outcome (PRO) measures is now a recognized standard of PSO practice in Canada [25,26,27,28]. Within a step-care model, PSO professionals are responsible for acknowledging PRO scores with patients, reviewing scores regularly, tracking patient progress over time, delivering evidence-based interventions within their scope of practice, and, with patients’ consent, facilitating necessary referrals to achieve measurable outcomes [26,27,28].

Other considerations: Maintaining a good balance between direct clinical practice and indirect workload responsibilities supports job satisfaction and reduces the risk of burnout among PSO professionals. Access to suitable workspace for privacy, along with occupational health and safety measures, contributes to professional well-being and job satisfaction [18]. Individual and/or group supervision, peer supervision, and case review rounds provide “checks and balances” in the system for quality care and support for PSO disciplines. Ongoing education and training opportunities enhance PSO professionals’ expertise and promote the delivery of evidence-based clinical practice [18].

### 3.2. Step 2: Defining the Scope of Practice

Discipline and Credentialing: The next step in the PSO staffing framework is to define the required discipline and credentialing. Understanding, rather than assuming, the scope of practice of a PSO discipline is an essential step in the hiring process. Scope of practice is determined by credentialing standards established by provincial regulatory bodies, and varies across Canadian provinces. For example, within the discipline of social work, credentials may include doctoral, masters, bachelors, and community college degrees or diplomas. In Ontario, psychotherapy is one of the fourteen controlled acts under the Regulated Health Professions Act, 1991, restricted to members of certain professions [29]. Licensed social workers may provide psychotherapy in compliance with the Social Work and Social Services Work Act of 1998, and its regulations and bylaws [29]. Provinces also legally regulate professional titles. For instance, in Ontario, registered social workers and social services workers are not permitted to use the title “clinical social worker” [29]. Administrators may consult professional practice leads within their organization and/or the provincial regulatory body to understand variations in credentialing, protected titles, and authorized practices.

Hired PSO professionals must hold a license in good standing. Those hired outside the province or country may practice under supervision while working towards licensure, usually negotiated as part of the contract. This measure ensures compliance with provincial standards, and professional accountability.

A clear understanding of PSO multidisciplinary scopes of practices is a foundation of the staffing framework. It highlights the value of PSO expertise in service delivery and its impact on the quality of life of people affected by cancer. This knowledge is essential for advocating and securing funding for PSO positions, and for transparently communicating core PSO professional competencies during recruitment and interviews. Several national organizations and cancer agencies have done the work of defining scopes of practice for various PSO professionals [19,23,24]. Reviewing, comparing, and adapting these documents to the local setting helps develop the full scope of practice required for the position.

The original CAPO staffing framework [18] focused on the discipline of social work. The scope of practice included the following: (1) initial assessment; (2) clinical interventions; (3) responding to distress scores within a stepped-care model; (4) specifying the types of services provided, e.g., for adults, couples, families, and children; (5) advocacy and instrumental work to secure necessary resources for patient care; (6) case conferencing and debriefing; (7) providing evidence-based interventions, specifically through transition periods across the cancer trajectory; (8) developing and/or facilitating group work; (9) teaching and mentoring; (10) program development and implementation; (11) leading and/or participating in research; and (12) complete administrative responsibilities [18]. When adapting the framework to other disciplines, responsibilities and tasks must reflect each profession’s scope of practice. Answering the questions in Appendix A supports adaptation of steps 1 and 2 of the staffing framework for other PSO disciplines. Identifying program strengths, service gaps, and areas of improvement provides a good foundation for determining appropriate discipline, credentialing, and scope of practice required to meet patient needs.

### 3.3. Step 3: Estimating the Number of Patients Requiring Services

Fitch’s [30] supportive care framework was developed to guide administrators and PSO professionals in understanding the types of support services cancer patients may require for effective service delivery planning. Its step-care model approach has shaped program planning and practice guideline development in Canada [26,31,32]. Under this framework, approximately 35% to 40% of cancer patients are projected requiring specialized PSO interventions to manage cancer-related distress symptoms [30]. For the CAPO staffing framework and formula [18], 35% was set as the minimum target rate of new cancer patients to be referred to a PSO program for counseling and/or psychotherapy. Tracking referral trends to the PSO program to understand the percentage of new cancer patients referred for PSO services is required for comparing with the 35% referral rate target set in the formula. When referral rates fall below 35%, strategies to increase awareness of PSO services with the cancer care team and within the community are necessary, including how patients can self-refer for PSO services. Providing education to team members about how to initiate and complete PSO referrals on patients’ behalf supports patients accessing services in time of need. If administrators are not seeing the expected referral rates to a PSO program, a deeper analysis may uncover barriers. Referral processes may be too cumbersome or unknown; screening for distress rates may have dropped; clinician capacity in clinics to prioritize the PSO needs of cancer patients is perhaps limited; or clinicians may not share the same values for addressing the PSO needs of cancer patients. Working collaboratively with clinicians, administrators and patient representatives in addressing these challenges is a means to find effective solutions.

If a discipline is required for a specialized clinic, the minimum 35% referral rate may not apply. For example, if all head and neck cancer patients generate automatic referrals to dietitians and speech language pathologists as part of the clinic standard, then the projected total number of patients will factor into the calculations of the staffing formula and not the expected minimum 35% referral rate. For the purpose of this paper, the 35% referral rate is part of the formula found in Appendix E—Step 10 to demonstrate how to calculate the total number of FTEs required for a cancer program.

### 3.4. Step 4: Defining Work Hours

Work hours vary across regions, provinces, and organizations, reflecting human resource policies, occupational health and safety standards, labor laws, and collective agreements. Clearly defining available work hours in a day is a requirement to estimate workload volume for one PSO provider. Workload volume exceeding available work hours compromises care and places unrealistic expectations on PSO providers, contributing to burnout.

The CAPO staffing formula calculations include the full-time equivalent hours of a position, paid time for non-working hours (statutory holidays, vacation), and planned daily breaks and lunches [18]. Hours for designated sick days and education days in a fiscal year also factor into the formula [18]. It does not include short-term and long-term disability leaves, unpaid leaves and/or family leaves, etc., as these absences fall outside the scope of predictability of expected time off for the average employee [18]. The breakdown of the calculations is found in Appendix B—Step 4 and can be adapted for any setting.

### 3.5. Step 5: Direct vs. Indirect Patient Care

Direct patient care (DPC) includes all activities required to deliver PSO services, such as reviewing initial referral information, reviewing the health record, consulting team members as necessary, planning interventions, meeting with patients (in person, telephone, or virtual), documenting, advocating, navigating the system, and ongoing required interdisciplinary consultations. Indirect patient care (IPC) includes non-clinical responsibilities, such as attending meetings; participating in rounds; entering workload data; working on policies, procedures, and standards; planning and developing programs; providing mentorship and student supervision; providing education/training; doing research; and other tasks as assigned by management/leadership.

Provincial agencies and/or regional programs often capture performance data based on a workload measurement system. In hospital settings, it is common to have a split of 80% DPC and 20% IPC, based on the distribution of responsibilities. It is widely accepted that if a health care professional is working above the 80% DPC threshold, then the program lacks human resources, with tremendous pressure placed on staff to achieve unattainable workload volume targets.

The 80–20% split ratio may not necessarily apply to outpatient or academic PSO programs, where responsibilities for education, research, project work, etc. are greater. In these settings, the split between DPC and IPC workload hours are adjustable to better reflect workload expectations; e.g., a 75% DPC-25% IPC split. To expect a PSO professionals to carry additional responsibilities while keeping the ratio of 80% DPC-20% IPC split is not a realistic workload volume target.

We demonstrate how to arrive at these percentages through a series of calculations in Appendix B—Step 5, using the CAPO example of a social worker working in a tertiary cancer center where the IPC activities are higher. We use the example of a 75–25% IPC split throughout Appendix C, Appendix D and Appendix E, Steps 6–10. We offer the example of a 70% DPC-30% IPC split in Appendix B—Step 5 and Table A1 in the manuscript, to demonstrate how to adjust the formula for calculations and compare allocation of work hours. What is important to retain is that IPC percentages increase based on various responsibilities held by PSO professionals in the program and may vary by discipline.

### 3.6. Step 6: Carryover Caseload from the Previous Year

Fiscal years vary by organization; a fiscal year will start 1 January and end 31 December, or it may start 1 April and run until 31 March. Regardless of the timeline, understanding how a caseload transfers from one fiscal year to the next is quite relevant in calculating the staffing formula. For example, when discharging a patient from an in-patient acute bed at the hospital, there is most likely no carryover caseload by members of the interdisciplinary team with the file closed. If the patient is receiving care in an outpatient cancer clinic, their file will most likely remain open across the cancer care trajectory. The staffing formula we propose captures the number of carryover cases from the previous fiscal year and the new cases for the entire new fiscal year.

In calculating the carryover caseload, it is imperative to estimate, as closely as possible, the number of unique open cases from the past year where patients will be seen again in the new fiscal year. If a patient is referred to a PSO program in February and continues to be followed by a PSO professional in April, at the beginning of the new fiscal year, the file remains open and is considered transferred to the next fiscal year. If a new file is open in February and the patient is only seen in that month, the case remains open but is not counted as part of the carryover caseload; only if the patient is seen again, say, in June, then the case is counted as part of the carryover caseload. Unfortunately, some programs consider all open cases as part of the carryover caseload regardless if patients receive services or not in the new fiscal year. This approach provides an unrealistic caseload for the PSO worker and makes it difficult for the administrator to gauge the anticipated work volume.

We know that patients return for PSO care if there are challenges with disease progression, if there is a new proposed treatment, or if there is a transition from treatment to survivorship. Most patients request continuity of care with the same PSO provider. The clinical notes should reflect that counseling/psychotherapy goals are met and that the patient is not returning for an appointment. The provider removes the patient’s name from their active caseload list and/or enters the status of the case file, opened or closed, in a workload measurement system.

There is limited data publicly available to understand the carryover caseload of PSO programs across Canada. For the purposes of this formula, the carryover caseload to the next fiscal year is 20% based on data made available by some administrators when the panel convened to develop the original CAPO hiring framework [18]. There was also consensus that each unique patient requires 4 h of service (based on 1 h per appointment), understanding that there may be variations. The one-hour appointment applies regardless of the service delivery model—in person, or by telephone or telehealth [18].

We understand that the 20% may not apply to other disciplines, based on some PSO professionals providing ongoing services to patient populations with certain types of diagnoses like head and neck cancers. Adjusting the 20% carryover caseload is feasible using the same calculation breakdown; we provide this in Appendix C—Step 6. If PSO programs and/or provincial agencies track and report data using these metrics, it may improve projection accuracy for carryover caseloads that matter for PSO human resource planning. The breakdown of the calculations to understand the workload volume for the carryover caseload to the next fiscal year is presented in Appendix C—Step 6.

### 3.7. Step 7: Distinction Between Interventions Provided and Allocation of Time

PSO professionals of the same discipline provide a variety of services that may differ based on their credentials and cancer program setting as described in Step 1. For example, social workers may provide counseling and/or psychotherapy, while also engaging in advocacy, navigation, and resource allocation activities [23,24]. Drawing on Fitch’s [30] supportive care framework, services are organized using a stepped care model that maximizes professional expertise. Within this model, all cancer patients should receive an orientation and educational materials helping them understand their treatments and access to available resources (e.g., peer-led support groups, transportation assistance, services available through not-for-profit cancer organizations, etc.) [30]. A subset of patients, however, still require the services of PSO professionals. For example, referrals to social workers are common for complex instrumental concerns that require advocacy and navigation for patient access to medication coverage, financial resources, special equipment, etc.

The CAPO staffing formula takes into consideration the allocation of time for counseling/psychotherapy and cases referred for complex instrumental concerns (e.g., practical, financial issues) to understand how the DPC hours are distributed [18]. Other disciplines adapting the formula to their profession are encouraged to undertake a similar analysis of the workload that requires a referral to a PSO professional based on their expertise. Work appropriately delegated to another discipline and/or professional maximizes the PSO professional’s time.

PSO programs may look at different models of care to deliver counseling and/or psychotherapy vs. instrumental and/or resource-related services. In small and mid-size cancer centers, MSW social workers may carry a greater proportion of counseling/psychotherapy cases and a smaller proportion of their caseload focused on instrumental concerns. In contrast, some cancer centers may employ BSW social workers to manage the large volume of cases with instrumental concerns, and hire MSW social workers [22] and/or psychologists to provide counseling/psychotherapy to patients with more complex psychosocial needs. The breakdown of responsibilities links directly back to steps 1 and 2 to understand the PSO program cancer model and scope of practices held by professionals. The CAPO staffing formula [18] incorporates time allocation for both counseling and/or psychotherapy and complex instrumental concern referrals to social workers. Other PSO disciplines are encouraged to apply the same process as part of adapting the staffing framework.

The breakdown of hours for assessments and follow-up sessions presented in Appendix C—Step 7 reflects consensus reached by panel members during the development of the CAPO staffing formula for the discipline of social work [18]. The estimate of six visits per patient is an average since individual needs vary, with some people requiring more visits, and others, fewer. These variations balance out over the fiscal year. The time allocation for the sessions with patients does not change regardless if the visit is in-person or via telephone or telehealth [18]. A workload measurement system is required to track the volume of caseloads and the distribution of new referrals over time, and to support transparent data reporting.

### 3.8. Step 8: Group Work

PSO team members offer a variety of group-based interventions, depending on the PSO program’s model of care. These typically include psycho-educational groups and therapeutic groups, available to patients during the year. Psychoeducational groups are generally open, single session, attended once, and repeated at different time intervals throughout the year. Therapeutic groups are usually closed groups, run over multiple weeks, and offered at different intervals annually. Group facilitation requirements vary by type of group, size, and clinical complexity, with some groups requiring one facilitator and others two facilitators. The work environment is fluid, and a well-developed workload measurement tool is essential to track, in real time, the volume of group work assigned to PSO providers.

Group facilitation affects individual caseload capacity. For example, if a PSO provider is not facilitating groups during the first quarter of a fiscal year, they may receive a higher volume of referrals for other types of counseling/psychotherapy, including individual, couple, family interventions, etc. If, by the next quarter, a PSO provider is facilitating groups, fewer appointment sessions are available for new individual, couple, or family referrals. Over the course of the year, these fluctuations tend to balance out. If group work is not included in a PSO provider’s scope of responsibilities during a fiscal year, additional DPC hours become available for other clinical activities/responsibilities.

The CAPO staffing formula [18] includes the time required to prepare for and facilitate a therapeutic group, as well as projected time allocated to group work when it runs repeatedly during a fiscal year. Calculations presented in Appendix D—Step 8 (Table A6**)** are adjustable to reflect discipline specific roles and responsibilities and time requirements.

### 3.9. Step 9: Calculating the Full Caseload of the PSO Professional

The calculated caseload of a PSO professional includes the availability of work hours for DPC and estimating the time for each activity: group work, counseling and/or psychotherapy, and instrumental concerns/advocacy work. It takes the total number of work hours calculated in step 4. It includes the DPC/ICP percentage splits to calculate the work hours for each stream of work as part of step 5. It includes the carryover caseload of Step 6. It includes how DPC hours are split based on interventions provided as part of Steps 7 and 8. Calculations of these hours using the formula produces projections for the total number of new patients a PSO provider can see in one year and the projected number of DPCCs. To illustrate the math to calculate the full caseload, we offer an example in Appendix E—Step 9 (Table A7), based on the CAPO staffing formula [18].

In summary, one FTE Social Worker providing three groups (*n* = 24 patients) per year, with remaining hours split between instrumental/advocacy @ 30% (*n* = 89 unique patients) and counseling and/or psychotherapy @ 70% (*n* = 96 unique patients), will see 209 new patients per year, with a carryover caseload of 60 unique patients, for a total caseload 269 unique patients and 1251 DPCCs in one fiscal year [18].

### 3.10. Step 10: Calculating the Number of PSO Positions for a Discipline—TertiaryCancer Center

To calculate the number of full-time equivalent (FTE) PSO positions required for a given discipline in a cancer program, identify the number of new cancer cases referred to the program in a fiscal year. Multiply this number by the prevalence of distress to calculate the expected volume of PSO referrals. The estimated workload volume of a PSO provider, following Steps 4–9, calculates the staffing ratio required to meet patient needs, as illustrated in Table A8. Variations in direct and indirect patient care splits (e.g., 75–25% split, 70–30% split, so on and so forth) change the number of FTEs required, as illustrated in Table 1.

## 4. Discussion

In this paper we describe a comprehensive staffing framework with a series of mathematical formulas to estimate staffing ratios for PSO programming. As a starting point, the work hours available reflect when the PSO provider is available for work, excluding vacation time, sick time, etc. Work hours are divided between DPC and IPC time. The total DPC hours are further divided into time spent on assessments and interventions provided by PSO professionals. These hours are combined into a mathematical formula to calculate the workload volume for one PSO provider. The final mathematical calculations include the estimated patient workload for one PSO provider, the unique number of cancer cases referred to a cancer program/center, and the minimum number of patients referred to a PSO program based on 35% prevalence of distress. Although previous publications reported staffing allocations for PSO programs, how they arrived at these calculations is unclear, making it difficult to compare them with the framework we propose.

Zebrack [21] reports a staffing ratio of 9.0 FTE social workers for a cancer center registering 3600 new cancer cases annually. Applying a 75% DPC–25% IPC time split, our staffing formula estimates a staffing ratio of 6.70 FTE social workers for a slightly larger cancer center (*n* = 4000), representing a difference of 2.3 FTEs, with implications for funding. However, our minimum distress level, for calculating the number of cases referred to a PSO program, is higher, at 35%, compared to their estimated distress level of 30% [21]. We should be reporting a greater number of FTE positions required based on more patients referred for PSO care. Several factors may account for these differences.

Firstly, Zebrack refers to “highly distressed” cancer patients as the primary population within social workers’ caseloads [21]. It is unclear whether this population includes a substantial proportion of palliative care patients, who are recognized to experience higher levels of emotional distress [33], which may partially explain the social workers’ reported caseload of 12 patients per month. The authors do not specify the number of other case types included in the social work caseload or describe additional responsibilities they may hold. Secondly, differences may reflect variations in the net available work hours after deducting vacation days, statutory holidays, etc. Thirdly, the allocation of work hours across workload responsibilities may also be a factor. For example, differences between a universally funded Canadian healthcare system and the non-universal Medicare system in the USA may result in social workers in the USA spending more time supporting people to access cancer care via insurance through navigation and advocacy, therefore increasing DPC time. Understanding the distribution of DPC and IPC hours, further broken down by DPC work activities, would help clarify observed differences in staffing ratios.

Making direct comparisons between our staffing ratios and the social work staffing ratios reported by Wilde et al. [22] is challenging. Specifically, the number of unique cancer cases referred across tertiary cancer centers, regional cancer centers, and community programs, or what proportion of cases are referred to PSO programs, is not reported in the paper [22]. Based on 2017 data, a 1.0 FTE social worker in a regional cancer center had a workload of 235 unique cancer patients (including group work) with 644 initial and follow-up contacts [22]. Our model estimates that a 1.0 FTE social worker has a caseload capacity for 209 new referrals annually, with a carryover caseload of 60 cases, a total of 269 unique patients with 1328 DPCCs.

The difference for workload capacity and DPCCs is understandable based on the percentages of time split between psychotherapy/counseling and instrumental/resource work. The regional cancer center social worker spent over 56% of their time doing instrumental/resource work if combining percentages spent on helping patients accessing cancer medications (8%), addressing financial concerns (34%), and assisting with travel/accommodation arrangements (14%) [22]. When combining advance care planning (4%) and support for family (6%) with counseling (29%), the overall time spent in counseling was 39%, with 5% designated for other work [22]. We report a 1.0 FTE social worker whose caseload is comprised of providing counseling/psychotherapy 75% of the time and instrumental/resource work 25% of the time. The increase in patient unique cases we report, *n* = 269, is possibly explained by the example we offer of a social worker offering three groups in a fiscal year. Participants referred for group work increase the DPCCs by capturing each person in attendance as a unique visit for the purposes of workload measurement. Group work is referenced in the Wilde et al. paper; however, the total number of participants is not reported [22]. We estimated eight participants per group, understanding that the number of participants fluctuates, possibly explaining the difference in workload volume. The DPCCs are also different: 644 DPCCs [25] vs. our calculations of 1251 DPCCs. The variations are possibly explained by how DPCCs are counted.

A number of factors may explain the variances if administrators find differences when comparing their staffing ratios to what we propose. If staffing ratios in PSO programs are higher than the estimates proposed in our framework, this is not an indication of overstaffing. A review for the allocation of total work hours, beginning with Step 4 of the model, is recommended. Understanding the allocation of DPC vs. IPC hours may explain the differences when IPC hours are higher. Referral rates below the assumed 35% threshold may indicate an opportunity to increase awareness of PSO services, and for referral rates to align with the 40% maximum level of distress proposed in Fitch’s model [30] for estimating the population requiring specialized psychosocial care. When the staffing ratios are similar, but the DPCCs differ, further analysis on how patient contacts are coded and captured in a workload measurement system is required. Lower contact numbers do not necessarily indicate underperformance by the PSO provider.

Given the limited number of administrative and policy-focused publications describing PSO staffing ratios in Canada, the staffing framework and formula we propose was developed from a consultation and consensus-building process with PSO leaders and clinicians across Canada. Its primary strength lies in the use of actual available work hours, divided between DPC and IPC activities. The systematic and structured approach to dividing the work hours, through a series of calculations for various interventions and workload responsibilities, provides a realistic workload and staffing requirements. The staffing framework and formula is adaptable to other PSO disciplines by following the steps outlined in this article.

## 5. Limitations

The example of the CAPO staffing framework referenced in this article for the discipline of social work reflects a generalist model of practice [18]. As such, it does not account for specialized clinics for stem cell transplants, head and neck cancers, palliative care clinics, etc. While the staffing framework remains applicable across these settings, the allocation of time per patient may need to increase to reflect the complex needs and intensity of care required by specific cancer populations. The allocation of time for assessment and intervention activities is adjustable based on the discipline(s) employed within specialized clinics. The minimum 35% referral rate for PSO programming may not apply if all patients within a clinic are referred to a PSO provider. Yet, the formula still applies by adjusting the numbers for every step.

Proposed staffing frameworks and ratios may vary across cancer centers due to differences in program maturity, infrastructure, and technology, all of which influence operational efficiencies. What mitigates the possibility of wide margins of discrepancies in staffing estimates is the reliance on total available work hours and the explicit description of how these hours are divided. Understanding the distribution between DPC and IPC work provides a starting point for comparing staffing ratios. The formula is fluid, allowing adjustments of time in the calculations, e.g., variations in the type of group work hours, assessment time, etc.

Cancer agencies reporting on the cancer system performance either provincially or federally are also positioned to collect and analyze data using our staffing framework and formula to set benchmarks as a means of understanding and addressing service gaps in PSO care. This analysis will go a long way to create an action plan to meet the psychosocial and supportive care needs of cancer patients and families requiring specialized interventions. This is feasible for every PSO discipline drawing on data from electronic workload measurement systems. Real-time documentation of patient encounters in electronic workload measurement tools, rather than reliance on estimated data, enhances the accuracy and quality of workload and staffing data.

The proposed staffing framework, which is adaptable to other PSO disciplines, does not address patient outcome measurements, as this fell outside the scope of the work. However, we argue that if models of PSO care are valued and funded based on the proposed staffing framework and ratios for all PSO disciplines, patient outcomes will become easier to measure. We call on governments to develop discipline-specific PSO indicators, beginning with the number of patients who access these services, to understand the gaps in PSO care across the country.

## 6. Conclusions

As models of cancer care evolve through advances in science and policy, so does our understanding of the psychosocial burden on individuals affected by cancer. Yet, the availability of equitable patient access to PSO programming and providers across Canada is lacking. A genuine commitment to person-centered cancer care requires an investment in the allocation of PSO resources.

The proposed staffing framework offers an objective method to assess and hire the required PSO discipline staff to meet the needs of people affected by cancer. Emphasis placed on staffing ratios for PSO disciplines providing services at the highest level of their scope of practice strengthens the interdisciplinary care provided to patients. Value placed on PSO workforce sustainability, achievable by promoting a well-balanced workload, enhances the quality of work life and retention for PSO professionals. The staffing framework and formula are adaptable to support the hiring of various disciplines across PSO and supportive care programming.

This work supports provincial, regional, and local health authorities in planning, developing, and implementing PSO staffing ratios for the chosen setting and required disciplines. PSO professionals and patient representatives may also use this framework to advocate for additional resources, particularly where current models of PSO care have not evolved, despite the evidence demonstrating an urgent need for accessibility to various specialized disciplines in PSO care. This is a call to action for healthcare administrators and policy makers across Canada to review their PSO programming and measure their staffing ratios against the staffing framework and formula we propose. CAPO remains open to collaborative work with governments reporting on the performance of the cancer system as a means to develop and track patient outcomes in PSO care. Cancer patients and their families require and deserve equitable access to specialized psychosocial care, regardless of where they live in Canada.

## Figures and Tables

**Table 1:** Number of FTE social work (SWRK) positions—Tertiary cancer center.

**Calculating Staffing Ratios**								
I. Based on 75% DPC-25% IDP Split Offering 3 Groups in 1 Fiscal Year								
a. New cancer cases referred to a tertiary cancer center	b. 35% prevalence of distress for new cancer cases (a × 35% = *n*)	c. SWRK caseload capacity for new referrals	d. SWRK FTE Ratio (b ÷ c = *n*)	e. Carryover unique cases from previous fiscal year	f. Total unique cases 1 SWRK (c + e = *n*)	g. Total DPCCs for new and carryover cases for 1 SWRK	h. Total SWRK unique cases for the PSO program (d × f = *n*)	i. Total SWRK DPCCs (d × g = *n*) for the PSO program
2000	700	209	3.35	60	269	1251	901	4191
3000	1050	209	5.02	60	269	1251	1350	6280
4000	1400	209	6.70	60	269	1251	1802	8382
5000	1750	209	8.37	60	269	1251	2252	10,471
6000	2100	209	10.05	60	269	1251	2704	12,573
II. Based on 70% DPC-30% IDP split offering 3 groups in 1 fiscal year								
2000	700	196	3.57	56	252	1175	900	4195
3000	1050	196	5.40	56	252	1175	1361	6345
4000	1400	196	7.10	56	252	1175	1789	8343
5000	1750	196	8.90	56	252	1175	2243	10,458
6000	2100	196	10.70	56	252	1175	2696	12,573

## Data Availability

The original contributions presented in this study are included in the article. Further inquiries can be directed to the corresponding author.

## Abbreviations

PSO	Psychosocial Oncology
SCO	Supportive Care Oncology
DPC	Direct Patient Care
IPC	Indirect Patient Care
DPCC	Direct Patient Care Contacts

## Appendix A

Steps 1 and 2: Staffing Framework and Formula

**Table A1:** Taking stock: infrastructure of the cancer program

Step 1: Hiring Principles What is the program setting? Tertiary, regional, community or other? What other disciplines are part of the PSO interdisciplinary team? ○ How do the roles overlap and complement each other? What disciplines form the larger interdisciplinary team? (E.g., physicians, pharmacists, nurses etc.) How developed is the program infrastructure? What administrative processes are in place to maximize efficiencies to support the PSO provider in the delivery of clinical care? ○ What quality improvement strategies are required? What processes and practices are in place in the cancer program to screen for distress? ○ What patient-reported outcome measures are completed by patients and/or family members? ○ What noticeable trends affect service delivery? Step 2: Defining Scope of Practice What is the discipline of the PSO provider? What credentials and licensure are required based on scope of practice? What types of assessments and interventions will they provide? What patient populations, defined by age, cancer type, and cancer trajectory, are served by the program? Other factors to consider: ○ Are family members eligible for PSO services? ○ Are there specialized clinics, and if so, how does it impact the decisions for hiring PSO professionals? ○ What evidence is available to understand the needs of the patient population receiving care and the type of PSO care required? What are the implications for credentialing and scope of practice of PSO health care professionals responding to distress scores in a step care model? Will the PSO provider hold academic, research, administrative, and/or program development responsibilities? ○ If yes, how many allocated hours per week to complete this work? ○ What supports are in place for the PSO provider? E.g., compensation, benefits and time off, regular clinical supervision, continuing education, confidential and safe space to work in, opportunities for professional mentoring, educational growth, etc.

## Appendix B

Step 4: Staffing Framework and Formula

**Table A2:** Calculating work hours

Step 4: Calculations (enter data for your own setting): 1 FTE = 1950 h (h) per year based on 37.5 h of work per week, 7.5 paid h per day Employees are usually at work 8 h per day; 0.5 h for unpaid lunch break Therefore, starting calculations at 7.5 h of paid work Assumes starting with 4 weeks of vacation: 37.5 h × 4 weeks = 150 h on average Assumes 12 statutory holidays: 12 × 7.5 h = 90 h on average 1950 h − 150 h (vacation) − 90 h (statutory holidays) = 1710 h of work available in 1 year 1710 of work hrs available ÷ 7.5 h = 228 days of work available 228 days × 0.5 h for 2 paid 15 min breaks per day = 114 h per year 1710 h of work − 114 h (breaks) = 1596 h of work available to continue with the calculation (7 h of work available per day)

Step 5: Staffing Framework and Formula

**Table A3:** Direct vs. indirect patient care hours

Step 5: Calculations (Enter the percentage split for your organization): Starting point: 1596 potential work h available (Table A2) 1596 potential work h × 75% DPC = 1197 h per year (171 days * per year) 7 h of work × 75% = 5.25 h of DPC per day 1596 potential work h × 25% IPC = 399 h per year (57 days * per year) 7 h of work × 25% = 1.75 h of IPC Or 1596 potential work h × 70% DPC = 1117 h per year (160 days * per year) 7 h of work × 70% = 4.9 h of DPC per day 1596 potential work h × 30% IPC = 479 h per year (68 days * per year) 7 h of work × 30% = 2.1 h of IPC

* Calculations based on 7.0 net h of work per day, as per Appendix B Table A2.

## Appendix C

Step 6: Staffing Framework and Formula

**Table A4:** Carryover caseload from one fiscal year to the next.

Step 6: Calculations: It is estimated that 20% of the possible work hrs will be directed to the carryover caseload for the next fiscal year for the discipline of social work Using the example of DPC 75-IPC 25% split from Table A3 1197 h of work available × 20% for the carryover caseload = 239 h 1197 h of work available − 239 h for carryover caseload = 958 work h remaining 239 h ÷ 4 h per unique case = 60 patients 60 patients with 4 DPCCs (Direct Patient Care Contacts) = 240 DPCCs 60 patients are part of the carryover caseload with 240 DPCCs Total work h remaining for DPC = 958 h; these hours are the starting point for the calculations in Step 7-Table A5 below

Step 7: Staffing Framework and Formula

**Table A5:** Distinction between interventions provided and allocation of time

Step 7: Calculations: Starting with 958 h of DPC available from calculations found in Table A4 Using the example of social workers focused primarily (70% of their time) on counseling/psychotherapy, with responsibilities (30% of their time) for instrumental concerns/resource allocation 958 h for DPC × 70% for counseling/psychotherapy = 671 h 958 h of DPC × 30% for instrumental concerns/resource allocation = 287 h Breakdown of hrs for counseling and/or psychotherapy: ○ New consult: 1.5 h ○ Follow-up 1 h × 5 visits = 5 h ○ 1 patient for counseling and/or psychotherapy = 6.5 h ○ 6 DPCCs for each patient Breakdown of h for instrumental concerns/resource allocation: ○ New consult: 1.0 h ○ Follow-up 1 h × 2 visits = 2 h ○ 1 patient for instrumental concerns = 3 h total ○ 3 DPCCs for each patient These hours factor into the calculation in Appendix E—Step 9

## Appendix D

Step 8: Staffing Framework and Formula

**Table A6:** Group work hours

Step 8: Calculations * Based on a social worker offering 1 therapeutic group per year ○ 8 participants joining a group (this may fluctuate) ○ 1/2 h per participant to screen = 4 h; 8 DPCCs ○ 3 h per group × 6 weeks = 18 work h (3 h broken down by the following workload activities: prepare content 0.5 h, set up room 0.5 h, facilitate the group 1.5 h, complete notes for the group 0.5 h) ○ 8 participants × 6 weeks = 48 DPCCs ○ Total worked h for 1 group: 4 + 18 = 22 h ○ Total DPCCs for 1 group: 8 DPCCs + 48 DPCCs = 56 DPCCs The formula in Appendix E—Step 9 includes the hours allocated for group work

* If more than one group is offered by the same PSO provider in a fiscal year, adjust the formula by using calculations for one group multiplied by the number of groups offered.

## Appendix E

Step 9: Staffing Framework and Formula

**Table A7:** Full caseload of PSO Professional in one year.

Step 9: Calculations: 958 work hours available (from Table A4.) Assumes 75% DPC-25% IPC split ○ Assumes the PSO provider offers 3 groups per year; 66 work h; 24 unique patients; 168 DPCCs. 958 work hrs available − 66 h for group work = 892 DPC h remaining Assumes 30% of the caseload is for instrumental concerns/advocacy (3 h per patient) ○ 892 h × 30% = 268 h; 268 h ÷ 3 h per patient = 89 unique patients ○ 89 unique patients × 3 visits = 267 DPCCs Assumes 70% of the caseload is for counseling and/or psychotherapy at 6.5 h per patient ○ 892 h × 70% = 624 h; 624 h ÷ 6.5 h = 96 unique patients ○ 96 unique patients × 6 DPCCs = 576 DPCCs Assumes 24 unique patients for group work + 89 unique patients for instrumental concerns + 96 patients for counseling and/or psychotherapy = 209 new unique patients ○ The workload volume of the social worker for new referrals in one fiscal year = 209 + the carryover caseload of 60 open cases from the previous fiscal year = 269 unique patients ○ 168 DPCCs for group work + 267 DPCCs for instrumental referrals + 576 DPCCs for counseling/psychotherapy = 1011 DPCCs ○ 1011 DPCCs + 240 DPCCs for the carryover caseload = 1251 DPCCs Summary: 1 FTE Social Worker providing 3 groups (*n* = 24 patients) per year with remaining hours split between instrumental/advocacy @ 30% (*n* = 89 unique patients) and counseling and/or psychotherapy @ 70% (*n* = 96 unique patients) will see 209 new patients per year with a carryover caseload of 60 unique patients for a total caseload 269 unique patients and 1251 DPCCs in one fiscal year.

Step 10: Staffing Framework and Formula

**Table A8:** Number of FTE PSO positions

Step 10: Calculations: New unique cancer patients referred to a cancer center; e.g., *n* = 2000 Distress @ 35% minimum of oncology patient population to be referred 2000 unique cancer patients × 35% distress = 700 patients require PSO specialized interventions A social worker can see 209 new patients based on a calculated caseload for new referrals in one fiscal year 700 potential new patients experiencing distress ÷ estimated social work caseload capacity of 209 = 3.3 FTE social workers
